# Salvage Ipilimumab plus Nivolumab after Anti-PD-1/PD-L1 Therapy in Advanced Hepatocellular Carcinoma

**DOI:** 10.1158/2767-9764.CRC-23-0072

**Published:** 2023-07-20

**Authors:** Stephanie L. Alden, Mir Lim, Chester Kao, Daniel Shu, Amit G. Singal, Anne Noonan, Paige Griffith, Marina Baretti, Won Jin Ho, Ihab Kamel, Mark Yarchoan, David Hsiehchen

**Affiliations:** 1 Sidney Kimmel Comprehensive Cancer Center, The Johns Hopkins University School of Medicine, Baltimore, Maryland.; 2 Department of Internal Medicine, University of Texas Southwestern Medical Center, Dallas, Texas.; 3 Department of Internal Medicine, The Ohio State University Wexner Medical Center, Columbus, Ohio.; 4 Department of Radiology and Radiological Science, The Johns Hopkins University School of Medicine, Baltimore, Maryland.

## Abstract

Combination anti-PD-(L)1/CTLA-4 blockade is approved in patients with hepatocellular carcinoma (HCC) in the first-line setting or after sorafenib, but whether this treatment has efficacy after prior anti-PD-(L)1 therapy is unknown. We performed a multicenter retrospective review of patients with advanced HCC treated with ipilimumab plus nivolumab after prior anti-PD-(L)1 therapy, excluding patients with prior anti-CTLA-4 treatment. Of the 32 patients who met our inclusion criteria, prior anti-PD-(L)1 regimens included atezolizumab plus bevacizumab (50%, *n* = 16), other anti-VEGF plus anti-PD-(L)1 combinations (31%, *n* = 10), and anti-PD-(L)1 monotherapy (19%, *n* = 6). The median number of prior systemic therapies was 2 (range, 1–8). The objective response rate with ipilimumab plus nivolumab by RECIST 1.1 was 22% [1 complete response (3%), 6 partial response (19%), 8 stable disease (25%), 16 progressive disease (50%), and 1 not evaluable (NE) (3%)], and objective response was associated with improved progression-free survival and overall survival. Immune-related adverse events were reported in 13 patients (41%), with no new safety signals. This study demonstrates that ipilimumab plus nivolumab has efficacy in patients with HCC who have received prior anti-PD-(L)1 therapy, suggesting that failure to respond to prior PD-(L)1 blockade should not preclude treatment with salvage ipilimumab plus nivolumab. Prospective studies are needed to define the optimal sequence of therapies.

**Significance:**

Anti-PD-(L)1 containing regimens are the preferred first-line treatment for advanced HCC, but whether salvage with PD-(L)1/CTLA-4 blockade is effective in patients who have failed prior anti-PD-(L)1 therapy is unknown. Our study demonstrates that ipilimumab plus nivolumab has clinical activity in patients with advanced HCC previously treated with anti-PD-(L)1 therapy, supporting the continued use of this regimen in the late-line setting after prior anti-PD-(L)1 exposure.

## Introduction

Hepatocellular carcinoma (HCC) is the seventh most common malignancy in the world but represents the second leading cause of cancer death (1, 2). Despite a recognizable at-risk population and effective HCC surveillance, up to half of patients diagnosed with HCC present with advanced, incurable disease (1, 2). Until recently, multi-kinase inhibitors were the only approved therapy for advanced, unresectable HCC, but recent clinical studies have established a role for immune checkpoint inhibitors, including anti-PD-(L)1 therapies, in this disease across multiple contexts (3–10).

On the basis of the phase III IMbrave150 trial, atezolizumab plus bevacizumab was the first immunotherapy combination regimen to show an improved overall survival (OS) benefit compared with sorafenib in the front-line setting for advanced HCC (3, 11). The efficacy of targeting CTLA-4 and PD-1 was shown by the CHECKMATE-040 study, demonstrating that ipilimumab plus nivolumab could induce durable responses in a subset of patients with HCC previously treated with sorafenib (4, 5, 12). More recently, the phase III HIMALAYA trial showed that tremelimumab plus durvalumab was associated with a significant improvement in OS compared with sorafenib in the first-line setting (10).

Thus far, all treatments for HCC after the first-line of therapy have been approved on the basis of trials enrolling patients intolerant or refractory to multi-kinase inhibitors, and, as a result, the optimal sequence of therapy for patients previously treated with immune checkpoint inhibitors is unknown. Prospective comparative effectiveness trials are unlikely to be conducted, so we will likely depend on retrospective series demonstrating efficacy and safety of different regimens in this setting.

While combined PD-(L)1/CTLA-4 blockade following anti-PD-(L)1 failure has demonstrated efficacy as a salvage regimen in other cancer types, it is unknown whether this treatment sequence is effective in HCC (13, 14). In particular, it is unclear whether CTLA-4 inhibition after progression on atezolizumab plus bevacizumab or other anti-VEGF plus anti-PD-(L)1 combinations have utility in HCC given that such regimens have been the preferred front-line treatments for eligible patients. Herein, we assess the clinical benefit and safety of ipilimumab plus nivolumab in patients previously treated with anti-PD-(L)1 therapy in HCC.

## Materials and Methods

### Design and Participants

Patients were identified on the basis of retrospective review of the electronic health record at the Johns Hopkins Hospital (July 1, 2016–November 15, 2022), University of Texas Southwestern Medical Center (March 1, 2020–November 1, 2022), and Parkland Health and Hospital System (March 1, 2020–November 1, 2022). Patient sex and race were obtained from the electronic health record. Eligible patients included all adults (18 years of age or older) with a diagnosis of HCC based on histology or imaging characteristics meeting American Association for the Study of Liver Diseases (AASLD) guideline criteria who had received at least one dose of anti-PD-(L)1 therapy with discontinuation for progression or alternative treatment, specified further below, followed by at least one dose of ipilimumab plus nivolumab (15). All patients had subsequent imaging and/or laboratory monitoring to determine disease progression. Patients with prior anti-CTLA-4 treatment or patients with fibrolamellar HCC, sarcomatoid HCC, or mixed cholangiocarcinoma/HCC were excluded. This study was approved by the Institutional Review Board at each of the respective sites and conducted in accordance with the Declaration of Helsinki.

### Treatment Regimens and Disease Monitoring

All patients received ipilimumab 3 mg/kg plus nivolumab 1 mg/kg, except for one patient who received ipilimumab 1 mg/kg plus nivolumab 3 mg/kg based on a concern for potential increased risk of immune-related adverse events (irAE) in the setting of underlying autoimmune disease. Patients received ipilimumab plus nivolumab every 3 weeks for up to four cycles followed by 480 mg nivolumab maintenance therapy every 4 weeks until disease progression, death, intolerance, or patient preference. Per routine practice at each site, patients had imaging every 6–12 weeks and laboratory monitoring prior to every cycle to determine disease progression. Radiographic response to treatment was assessed using cross-sectional imaging per RECIST v1.1. In 4 patients, clinical progression was determined by the treating physician based on signs or symptoms consistent with progressive disease (PD) and a concomitant rise in alpha-fetoprotein levels (16).

### Statistical Analysis

The primary outcome in our study was the objective response rate (ORR), defined as patients who achieved complete response (CR) or partial response (PR). The secondary outcome variables in our study were progression-free survival (PFS), calculated as the time from the first dose of ipilimumab plus nivolumab to disease progression or death from any cause, and OS, calculated as the time from the first dose of ipilimumab plus nivolumab to death from any cause. We also evaluated the duration of response (DOR), defined as the time from the first dose of ipilimumab plus nivolumab to disease progression in patients who achieved a CR or PR. irAEs were graded using the NCI Common Terminology Criteria for Adverse Events v4.0.

All analyses were performed using R (V4.2.1), with the survival and survminer packages used to calculate PFS, OS, and DOR. Continuous variables were described with median, range, number, and percentage of the cohort. Differences among categorical variables were assessed using Fisher exact test. Median PFS, OS, and DOR were estimated using Kaplan–Meier analyses with log-rank test used to assess differences in PFS and OS across subgroups of interest, including response (responders vs. nonresponders), body mass index (BMI; overweight/obese vs. normal/underweight), and HCC etiology (viral vs. nonviral). A *P* value < 0.05 was considered statistically significant for all analyses.

### Data Availability Statement

The data generated in this study are not publicly available to preserve patient confidentiality and due to the Health Insurance Portability and Accountability Act, however, deidentified data are available upon reasonable request from the corresponding author.

### Role of the Funder/Sponsor

The funder had no role in the design and conduct of the study; collection, management, analysis, and interpretation of the data; preparation, review, or approval of the article; and decision to submit the article for publication.

## Results

### Patient Demographics

We identified 32 patients with advanced HCC and prior anti-PD-(L)1 therapy who were subsequently treated with ipilimumab plus nivolumab (Table 1). The median age was 67 years, 59% (*n* = 19) of patients had viral-associated cirrhosis, with 47% (*n* = 15) due to hepatitis C and 13% (*n* = 4) due to hepatitis B; 66% (*n* = 21) of patients had Child-Pugh (CP) A disease prior to initiating ipilimumab plus nivolumab. With respect to Barcelona Clinic Liver Cancer (BCLC) stage, 13% (*n* = 4) of patients were stage B and 88% (*n* = 28) were stage C at the time of treatment.

**TABLE 1:** Patient demographics^a^

Clinical values	Overall cohort (*n* = 32)
Median age (range), years	67.0 (43.0–80.0)
Sex, no. (%)	
Male	28 (88%)
Female	4 (13%)
Race, no. (%)	
White	16 (50%)
African American	13 (41%)
Asian	3 (9%)
BCLC stage, no. (%)	
Stage B	4 (13%)
Stage C	28 (88%)
Etiology, no. (%)	
Viral	19 (59%)
HCV	15 (47%)
HBV	4 (13%)
Alcohol	1 (3%)
NAFLD	9 (28%)
Other	3 (9%)
Median BMI (range), kg/m^2^	26.2 (19.1–39.6)
ECOG, no. (%)	
0–1	27 (84%)
2+	5 (16%)
Child Pugh at start of treatment, no. (%)	
A	21 (66%)
B	11 (34%)
Extrahepatic spread, no. (%)	20 (63%)
Number of prior systemic treatments, no. (%)	
1	10 (31%)
2	15 (47%)
3+	7 (22%)
Prior ICI treatments, no. (%)	
Atezolizumab plus bevacizumab	16 (50%)
Other VEGF plus ICI combo	10 (31%)
ICI monotherapy	6 (19%)

Abbreviations: BMI, body mass index; BCLC, Barcelona Clinic Liver Cancer; ECOG, European Cooperative Oncology Group; HBV, hepatitis B virus; HCV, hepatitis C virus; ICI, immune checkpoint inhibitor; NAFLD, non-alcoholic fatty liver disease; VEGF, vascular endothelial growth factor.

^a^Select percentages may not add up to 100% due rounding.

Prior to treatment with ipilimumab plus nivolumab, 31%, 47%, and 22% of patients received one, two, and three or more lines of systemic treatments, respectively (Table 1). Prior lines of anti-PD-(L)1 therapy included immunotherapy doublet regimens comprised of atezolizumab plus bevacizumab (50%, *n* = 16), other anti-VEGF plus anti-PD-(L)1 combination therapies (31%, *n* = 10), and anti-PD-(L)1 monotherapy (19%, *n* = 6).

There were 28 patients (88%) who discontinued anti-PD-(L)1 therapy due to disease progression. Three patients (9%) stopped therapy for planned surgical resection after 2 months of anti-PD-(L)1 therapy. One patient (3%) stopped therapy due to lack of response to anti-PD-(L)1 therapy plus grade 2 esophagitis, managed with oral steroids, with transition to a multi-kinase inhibitor. The best overall response (BOR) to prior PD-(L)1 blockade was PD in 56% of patients (*n* = 18), stable disease (SD) in 38% of patients (*n* = 12), and PR in 6% of patients (*n* = 2). Of note, the 4 patients who discontinued therapy for surgical resection and lack of response to therapy had a BOR of SD.

### Treatment Outcomes with Ipilimumab plus Nivolumab

**FIGURE 1:**
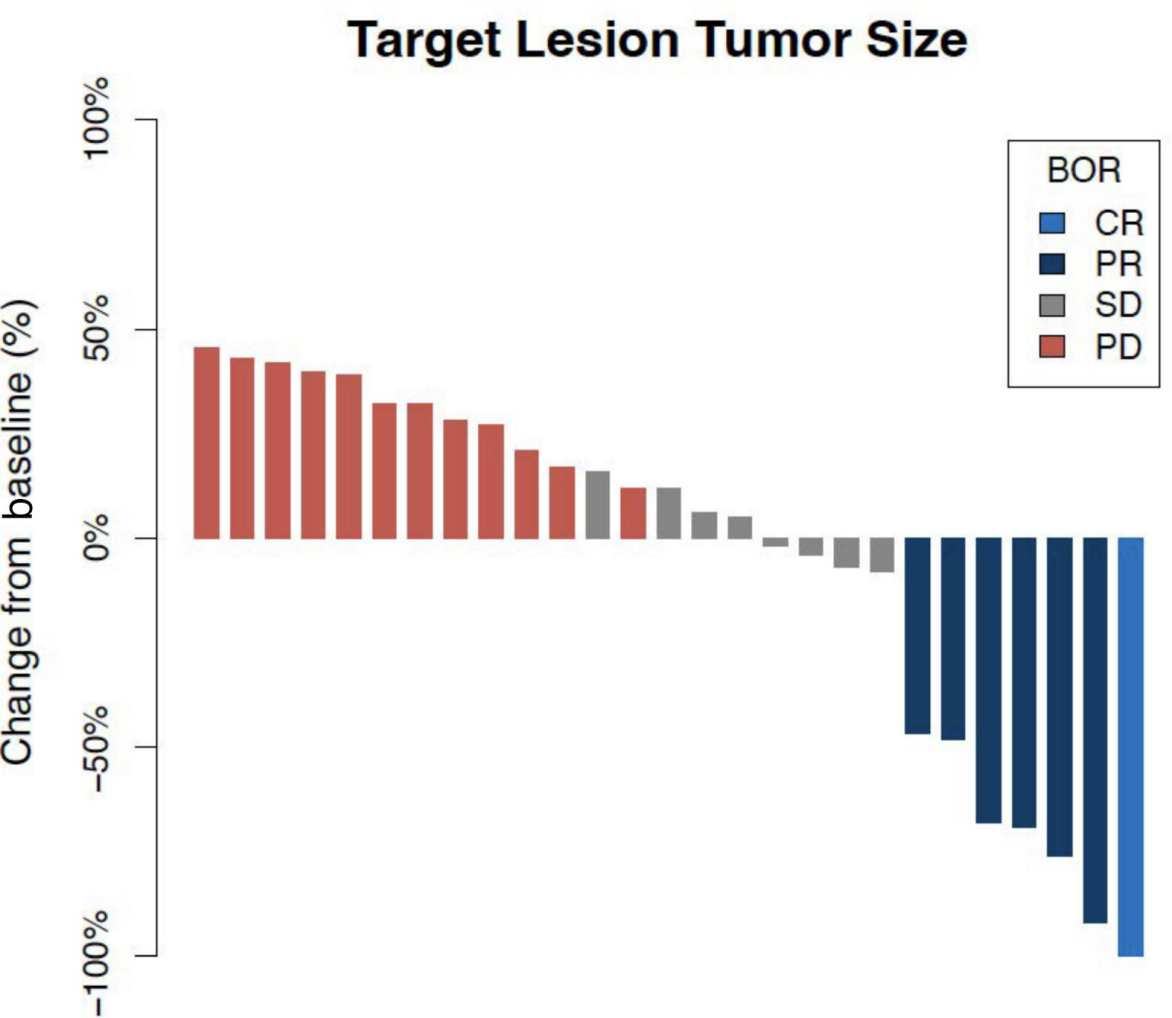
Target lesion change and objective response. Percent change in patient target lesion(s), determined by investigator review of pretreatment and posttreatment imaging, with BOR using criteria from RECIST v1.1. BOR, best overall response; CR, complete response; PD, progressive disease; PR, partial response; SD, stable disease.

The ORR with ipilimumab plus nivolumab after prior anti-PD-(L)1 therapy was 22%, including 1 patient (3%) with a CR and 6 patients (19%) with a PR. In addition, 8 patients demonstrated SD (25%), for a disease control rate of 47% (Fig. 1). One patient's response was not evaluable, as they developed a terminal irAE prior to response assessment, and an additional 16 patients (50%) had PD.

Among patients with an objective response to salvage ipilimumab plus nivolumab, none had an objective response to prior anti-PD-(L)1. Objective response was seen in 3 of the 12 patients who previously demonstrated SD and 4 of the 18 patients who previously demonstrated PD. Among the 4 patients who discontinued therapy for surgical resection and lack of response to therapy, 2 had an objective response to ipilimumab plus nivolumab, with both patients discontinuing first-line anti-PD-(L)1 therapy for surgical resection. There was no difference in BOR based on BMI (overweight/obese vs. normal/underweight, *P* = 0.22) or HCC etiology (viral vs. nonviral, *P* = 0.09).

**FIGURE 2:**
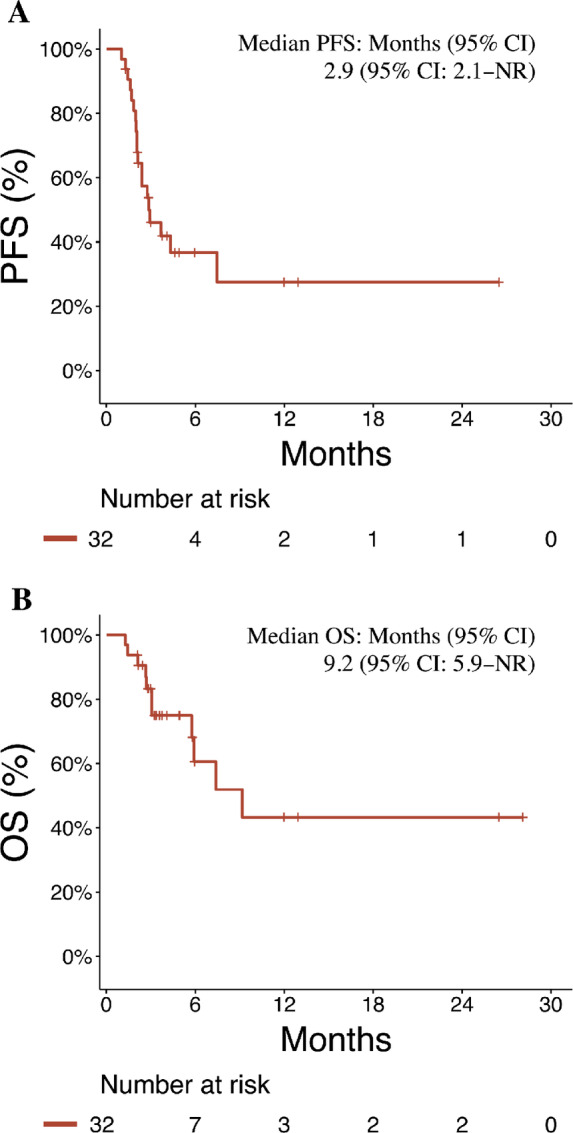
Survival characteristics. PFS (**A**) and OS (**B**) for the overall cohort, in months. CI, confidence interval; NR, not reached.

The median PFS was 2.9 months [95% confidence interval (CI): 2.1–not reached], and the median OS was 9.2 months (95% CI: 5.9–not reached; Fig. 2A and B). The DOR in patients who responded to ipilimumab plus nivolumab was 6.0 months (95% CI: 4.1–not reached). Objective response to ipilimumab plus nivolumab was associated with improved PFS (2.4 months vs. not reached, *P* = 0.004) and OS (5.9 months vs. not reached, *P* = 0.02; Supplementary Fig. S1A and S1B). There were no statistically significant differences in PFS and OS after salvage ipilimumab plus nivolumab when stratifying patients by BMI or HCC etiology (Supplementary Table S1).

### irAEs

During patients’ first line of anti-PD-(L)1 therapy, 7 (22%) experienced irAEs, including grade 2 hypothyroidism (*n* = 3, 9%), grade 2 hyperthyroidism (*n* = 1, 3%), grade 2 esophagitis (*n* = 1, 3%), grade 1 dermatitis (*n* = 1, 3%), and grade 1 transaminitis (*n* = 1, 3%). Among patients who experienced irAEs with the first line of anti-PD-(L)1, 3 (9%) experienced irAEs when treated with ipilimumab plus nivolumab. These 3 patients included: a patient who experienced grade 2 esophagitis, who then discontinued ipilimumab plus nivolumab due to grade 4 colitis; a patient who experienced grade 2 hyperthyroidism, who experienced grade 3 pneumonitis, grade 1 esophagitis, and grade 2 inflammatory arthritis with ipilimumab plus nivolumab, all managed with steroids, allowing the patient to continue nivolumab maintenance; and 1 of the 3 patients who experienced grade 2 hypothyroidism, who developed grade 5 hepatitis, as detailed below.

During treatment with ipilimumab plus nivolumab, irAEs were more frequent as compared with the first line of anti-PD-(L)1 therapy, as expected. irAEs occurred in 13 patients (41%) during salvage ipilimumab plus nivolumab (Table 2). The most common irAE was pneumonitis (*n* = 4, 13%), followed by colitis (*n* = 3, 9%), rash (*n* = 3, 9%), and hepatitis (*n* = 3, 9%). A grade 5 treatment-related adverse event of autoimmune hepatitis was observed in 1 (3%) patient. This fatal irAE occurred after two doses of therapy in a patient with CP A5 cirrhosis. Grade 3–4 irAEs were observed in 6 (19%) patients. Among patients who developed irAEs with ipilimumab plus nivolumab, 5 discontinued treatment due to toxicity, including 1 patient with Crohn disease who received flipped dose ipilimumab plus nivolumab. Two of the 5 patients were rechallenged with ipilimumab plus nivolumab: 1 patient developed recurrent autoimmune hepatitis, requiring permanent discontinuation of therapy, while the other had no further complications. Neither of these 2 patients experienced irAE with prior anti-PD-(L)1 therapy. Notably, this regimen was found to be safe in patients with CP B cirrhosis, with no increased risk of irAE in this population.

**TABLE 2:** irAEs with ipilimumab plus nivolumab^a^

Event	Grade 1–2	Grade 3–4	Grade 5	Total (*n* = 32)
Pneumonitis	2 (6%)	2 (6%)	0	4 (13%)
Colitis/esophagitis	2 (6%)	2 (6%)	0	4 (13%)
Autoimmune hepatitis	1 (3%)	1 (3%)	1 (3%)	3 (9%)
Rash	3 (9%)	0	0	3 (9%)
Arthritis	1 (3%)	0	0	1 (3%)
Myocarditis	0	1 (3%)	0	1 (3%)

^a^Select percentages may not add up to 100% due rounding.

## Discussion

This study demonstrates that salvage ipilimumab plus nivolumab in HCC is associated with clinically meaningful objective response and disease control. In addition, objective responses with ipilimumab plus nivolumab were achieved in patients with primary resistance to anti-PD-(L)1 therapy, adding to growing preclinical and clinical evidence that combined PD-(L)1/CTLA-4 blockade can overcome PD-(L)1 resistance in HCC and other tumor types (10, 13). The response rate observed in this study (22%) was somewhat lower than that reported for ipilimumab plus nivolumab in the prospective Checkmate 040 study (32%; refs. 4, 5, 10). However, this may reflect the immune checkpoint refractory population in this study, as the Checkmate 040 study enrolled patients with no prior exposure to anti-PD-(L)1 therapy. There were no discernable differences in safety or efficacy of salvage ipilimumab plus nivolumab across patient subsets such as nonviral or viral etiologies, but this requires further confirmation, as our study is likely underpowered to detect weak associations.

This retrospective study was conducted in the setting of a rapidly evolving treatment landscape for advanced HCC. At the time of this report, bevacizumab plus atezolizumab is the preferred first-line therapy for patients with advanced HCC, and there is little available data to guide treatment decisions in subsequent therapy lines (15, 17, 18). On the basis of expert consensus opinion, clinical guidelines from AASLD, American Society of Clinical Oncology, National Comprehensive Cancer Network (NCCN), and others recommend the use of multi-target tyrosine kinase inhibitors in patients progressing on bevacizumab plus atezolizumab, but some patients may not be candidates for such therapies, including patients with bleeding events on bevacizumab plus atezolizumab (15, 17, 18). Our retrospective data suggest that ipilimumab plus nivolumab is a treatment option for patients with prior exposure to anti-PD-(L)1 therapy and can be effective even in patients who do not respond to bevacizumab plus atezolizumab or other anti-PD-(L)1–based regimens.

This study shows that a lack of response to prior anti-PD-(L)1 therapy does not preclude a subsequent response to combined PD-(L)1/CTLA-4 blockade, suggesting that CTLA-4 blockade enhances antitumor immunity and/or activates immune cell programs unaffected by anti-PD-(L)1 monotherapy (10, 13). While targeting either immune checkpoints may enhance the immune system's antitumor response, they do so via different mechanisms. CTLA-4 expressed on T cells blunts the immune system's response to tumor antigens by blocking binding of B7 on antigen-presenting cells (APC) to CD28 on T cells, resulting in suppression of APC function and impaired activation of CD8^+^ T cells (19). The interaction between PD-1 on cytotoxic lymphocytes and PD-L1 on APCs and tumor cells transmits an inhibitory signal that induces CD8^+^ T-cell apoptosis and prevents T cell–mediated apoptosis (19–21). This mechanism is consistent with analyses of melanoma tissue after treatment with either anti-PD-(L)1 monotherapy, anti-CTLA-4 monotherapy, or in combination, which show distinct gene expression profiles associated with each type of treatment (22).

Salvage ipilimumab plus nivolumab was associated with irAEs in a subset of patients, with no new safety signals observed. irAE rates in this cohort were lower than those seen in prospective trials evaluating combined PD-(L)1/CTLA-4 blockade with ipilimumab plus nivolumab in HCC (4, 5, 10). This finding is potentially attributable to selection bias, as treating physicians likely only considered patients for salvage ipilimumab plus nivolumab in the setting of prior anti-PD-(L)1 therapy if they had tolerated prior immune checkpoint inhibition. Despite this finding, the single case of fatal hepatitis in our retrospective cohort underscores the potential toxicity and need for careful monitoring with ipilimumab plus nivolumab in any line of therapy.

### Limitations

Despite being the largest study to date that has evaluated outcomes of combined PD-L(1)/CTLA-4 blockade in HCC after failing anti-PD-(L)1 therapy, our cohort is small, which limits the generalizability of our findings and our ability to compare patient outcomes based on important clinical and demographic factors. In addition, while all patients received at least one form of anti-PD-(L)1 therapy prior to ipilimumab plus nivolumab, they received different types and numbers of systemic treatments, making our patient population heterogeneous. Future studies should include larger populations with more stringent inclusion criteria to better identify factors that influence response to treatment.

In our cohort, ipilimumab plus nivolumab resulted in ORRs comparable with those seen in other studies of anti-PD-(L)1/CTLA-4 combination therapy. There was an acceptable safety profile with no new safety signals; however, we did observe one grade 5 irAE of autoimmune hepatitis. Overall, these findings suggest that salvage ipilimumab plus nivolumab has meaningful clinical activity in select patients with advanced HCC who failed prior anti-PD-(L)1 therapy, including regimens incorporating anti-VEGF agents. Larger prospective studies are warranted to better define the optimal sequence of therapy in advanced HCC.

## Supplementary Material

Figure S1 Figure S1. Survival Characteristics by Response

Table S1 Table S1. PFS and OS based on Clinical Characteristics

## References

[1] Park J , Chen M, Colombo M, Roberts LR, Schwartz M, Chen P-J, . Global patterns of hepatocellular carcinoma management from diagnosis to death: the BRIDGE study. Liver Int 2015;35:2155–66. 25752327 10.1111/liv.12818 PMC4691343

[2] McGlynn KA , Petrick JL, El-Serag HB. Epidemiology of hepatocellular carcinoma. Hepatology 2021;73:4–13. 10.1002/hep.31288 PMC7577946 32319693

[3] Finn RS , Qin S, Ikeda M, Galle PR, Ducreux M, Kim T-Y, . Atezolizumab plus bevacizumab in unresectable hepatocellular carcinoma. N Engl J Med 2020;382:1894–905. 32402160 10.1056/NEJMoa1915745

[4] Yau T , Kang YK, Kim TY, El-Khoueiry AB, Santoro A, Sangro B, . Efficacy and safety of nivolumab plus ipilimumab in patients with advanced hepatocellular carcinoma previously treated with sorafenib: the CheckMate 040 randomized clinical trial. JAMA Oncol 2020;6:e204564. 33001135 10.1001/jamaoncol.2020.4564 PMC7530824

[5] El-Khoueiry AB , Sangro B, Yau T, Crocenzi TS, Kudo M, Hsu C, . Nivolumab in patients with advanced hepatocellular carcinoma (CheckMate 040): an open-label, non-comparative, phase 1/2 dose escalation and expansion trial. Lancet 2017;389:2492–502. 28434648 10.1016/S0140-6736(17)31046-2 PMC7539326

[6] Yau T , Park JW, Finn RS, Cheng AL, Mathurin P, Edeline J, . Nivolumab versus sorafenib in advanced hepatocellular carcinoma (CheckMate 459): a randomised, multicentre, open-label, phase 3 trial. Lancet Oncol 2022;23:77–90. 34914889 10.1016/S1470-2045(21)00604-5

[7] Finn RS , Ryoo BY, Merle P, Kudo M, Bouattour M, Lim HY, . Pembrolizumab as second-line therapy in patients with advanced hepatocellular carcinoma in KEYNOTE-240: a randomized, double-blind, phase III trial. J Clin Oncol 2020;38:193–202. 31790344 10.1200/JCO.19.01307

[8] Zhu AX , Finn RS, Edeline J, Cattan S, Ogasawara S, Palmer D, . Pembrolizumab in patients with advanced hepatocellular carcinoma previously treated with sorafenib (KEYNOTE-224): a non-randomised, open-label phase 2 trial. Lancet Oncol 2018;19:940–52. 29875066 10.1016/S1470-2045(18)30351-6

[9] Llovet JM , Ricci S, Mazzaferro V, Hilgard P, Gane E, Blanc JF, . Sorafenib in advanced hepatocellular carcinoma. N Engl J Med 2008;359:378–90. 18650514 10.1056/NEJMoa0708857

[10] Abou-Alfa GK , Lau G, Kudo M, Chan SL, Kelley RK, Furuse J, . Tremelimumab plus durvalumab in unresectable hepatocellular carcinoma. NEJM Evid 2022;1. 10.1056/EVIDoa2100070 38319892

[11] Casak SJ , Donoghue M, Fashoyin-Aje L, Jiang X, Rodriguez L, Shen YL, . FDA approval summary: atezolizumab plus bevacizumab for the treatment of patients with advanced unresectable or metastatic hepatocellular carcinoma. Clin Cancer Res 2021;27:1836–41. 33139264 10.1158/1078-0432.CCR-20-3407

[12] Saung MT , Pelosof L, Casak S, Donoghue M, Lemery S, Yuan M, . FDA approval summary: nivolumab plus ipilimumab for the treatment of patients with hepatocellular carcinoma previously treated with sorafenib. Oncologist 2021;26:797–806. 33973307 10.1002/onco.13819 PMC8417871

[13] Larkin J , Chiarion-Sileni V, Gonzalez R, Grob JJ, Cowey CL, Lao CD, . Combined nivolumab and ipilimumab or monotherapy in untreated melanoma. N Engl J Med 2015;373:23–34. 26027431 10.1056/NEJMoa1504030 PMC5698905

[14] Gul A , Stewart TF, Mantia CM, Shah NJ, Gatof ES, Long Y, . Salvage ipilimumab and nivolumab in patients with metastatic renal cell carcinoma after prior immune checkpoint inhibitors. J Clin Oncol 2020;38:3088–94. 32491962 10.1200/JCO.19.03315 PMC7499610

[15] Singal A , Llovert J, Yarchoan M, Mehta N, Heimbach JK, Dawson LA, . AASLD guidance on prevention, diagnosis and treatment of hepatocellular carcinoma. Hepatology 2023 [Online ahead of print]. 10.1097/HEP.0000000000000466 PMC10663390 37199193

[16] Fournier L , Ammari S, Thiam R, Cuénod CA. Imaging criteria for assessing tumour response: RECIST, mRECIST, Cheson. Diagn Interv Imaging 2014;95:689–703. 24951349 10.1016/j.diii.2014.05.002

[17] Gordan JD , Kennedy EB, Abou-Alfa GK, Beg MS, Brower ST, Gade TP, . Systemic therapy for advanced hepatocellular carcinoma: ASCO guideline. J Clin Oncol 2020;38:4317–45. 33197225 10.1200/JCO.20.02672

[18] NCCN Clinical Practice Guidelines in Oncology (NCCN Guidelines®). NCCN Guidelines Versions 2. 2022 Hepatobiliary Cancers. Published online January 8, 2023.

[19] Kudo M . Scientific rationale for combination immunotherapy of hepatocellular carcinoma with anti-PD-1/PD-L1 and anti-CTLA-4 antibodies. Liver Cancer. 2019;8:413–26. 32479569 10.1159/000503254 PMC6883444

[20] Alsaab HO , Sau S, Alzhrani R, Tatiparti K, Bhise K, Kashaw SK, . PD-1 and PD-L1 checkpoint signaling inhibition for cancer immunotherapy: mechanism, combinations, and clinical outcome. Front Pharmacol 2017;8:561. 28878676 10.3389/fphar.2017.00561 PMC5572324

[21] Shi F , Shi M, Zeng Z, Qi RZ, Liu ZW, Zhang JY, . PD-1 and PD-L1 upregulation promotes CD8+ T-cell apoptosis and postoperative recurrence in hepatocellular carcinoma patients. Int J Cancer 2011;128:887–96. 20473887 10.1002/ijc.25397

[22] Das R , Verma R, Sznol M, Boddupalli CS, Gettinger SN, Kluger H, . Combination therapy with anti–CTLA-4 and anti–PD-1 leads to distinct immunologic changes *in vivo*. J Immunol 2015;194:950–9. 25539810 10.4049/jimmunol.1401686 PMC4380504

