# Supplementary material for: Salvage Ipilimumab plus Nivolumab after Anti-PD-1/PD-L1 Therapy in Advanced Hepatocellular Carcinoma
Source: Cancer Res Commun. 2023 Jul 20;3(7):1312–7. doi: 10.1158/2767-9764.CRC-23-0072 (PMC10356567; doi:10.1158/2767-9764.CRC-23-0072)
Supplement: Figure S1 — Survival Characteristics by Response [file crc-23-0072-s01.pdf]

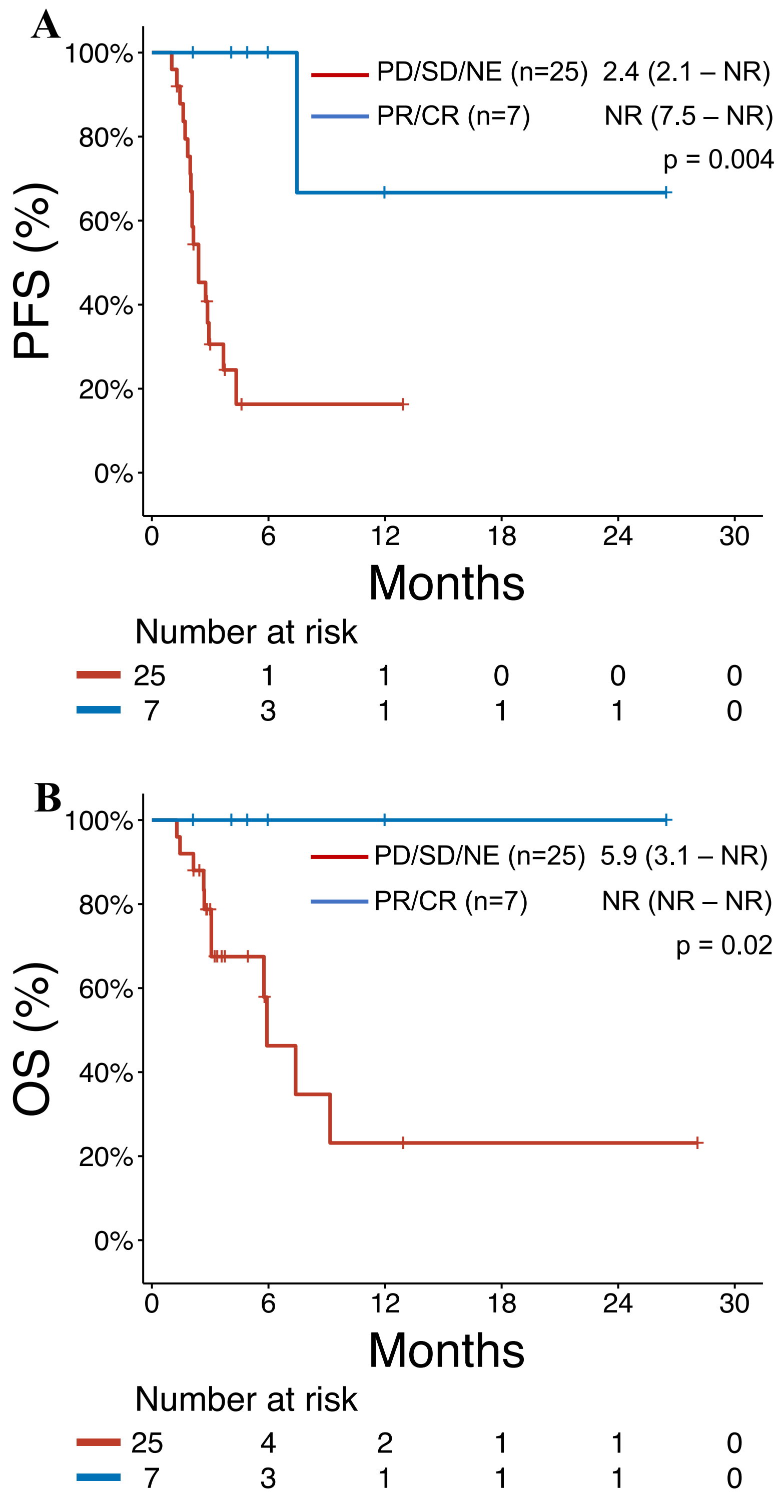

Figure S1. Survival Characteristics by Response
Patients without an objective response to ipilimumab plus nivolumab had significant differences in (**A**) PFS and (**B**) OS.
Abbreviations: PFS (progression free survival); OS (overall survival); SD (stable disease); PD (progressive disease); PR (partial response); CR (complete response); NR (not reached).
