# Supplementary material for: Salvage Ipilimumab plus Nivolumab after Anti-PD-1/PD-L1 Therapy in Advanced Hepatocellular Carcinoma
Source: Cancer Res Commun. 2023 Jul 20;3(7):1312–7. doi: 10.1158/2767-9764.CRC-23-0072 (PMC10356567; doi:10.1158/2767-9764.CRC-23-0072)
Supplement: Table S1 — PFS and OS based on Clinical Characteristics [file crc-23-0072-s02.pdf]

Table S1. PFS and OS based on Clinical Characteristics

Clinical Characteristic	PFS, Median (95% CI), months	p-value	OS, Median (95% CI), months	p-value
Obese/overweight vs. normal/underweight	3.7 (2.1-NR) vs. 2.4 (2.1-NR)	0.4	NR (5.9-NR) vs. 5.8 (3.1-NR)	0.2
Viral vs. non-viral etiology	2.9 (2.1-NR) vs. 3.7 (2.1-NR)	0.6	5.9 (5.8-NR) vs. NR (9.2-NR)	0.4

PFS (progression free survival); OS (overall survival); CI (confidence interval); irAE (immune related adverse event); NR (not reached); OR (objective response); PD-1 (programmed cell death protein 1); PD-L1 (programmed cell death ligand 1)
